# The Prognostic Role of the Class III β-Tubulin in Non-Small Cell Lung Cancer (NSCLC) Patients Receiving the Taxane/Vinorebine-Based Chemotherapy: A Meta-Analysis

**DOI:** 10.1371/journal.pone.0093997

**Published:** 2014-04-04

**Authors:** Yan-Long Yang, Xiu-Ping Luo, Lei Xian

**Affiliations:** 1 Department of Cardiothoracic Surgery, the First Affiliated Hospital of Guangxi Medical University, Nanning, Guangxi Zhuang Autonomous Region, China; 2 Clinical Faculty of Guangxi Medical University, Nanning, Guangxi Zhuang Autonomous Region, China; University of Kentucky College of Medicine, United States of America

## Abstract

**Background:**

A number of studies have examined the relationship between the expression of the class III β-tubulin (TUBB3) and the treatment responses to the taxane/vinorebine-based chemotherapy in patients with non-small cell lung cancer (NSCLC). However, the results of these studies were inconsistent and inconclusive. Therefore, we conducted an up-to-date meta-analysis to evaluate the prognostic role of TUBB3 in the taxane/vinorebine-based chemotherapy.

**Methods:**

A literature search for relevant studies was conducted in PubMed, Embase, and CNKI. The inclusion criteria were the taxane/vinorebine-based chemotherapy in patients with NSCLC and the evaluation of the clinical outcomes in relation to the expression of TUBB3. The clinical outcomes analyzed in this study included the overall response rate (ORR), overall survival (OS), and event-free survival (EFS). Odds ratio (OR) or hazard ratio (HR) with 95% confidence interval (CI) were calculated to assess the risk associated with the TUBB3 expression in the taxane/vinorebine-based chemotherapy.

**Results:**

A total of 28 studies with 2401 NSCLC patients were qualified for this meta-analysis. We found that the positive or high level of TUBB3 expression was associated with a poorer ORR (OR = 0.24, 95% CI = 0.16–0.36, *p*<0.001), an unfavorable OS (HR = 1.52, 95% CI = 1.27–1.82, *p*<0.001), and a worse EFS (HR = 1.47, 95% CI = 1.24–1.74, *p*<0.001) compared to the negative or low level of TUBB3 expression. The statistically significant associations between TUBB3 and chemotherapy responses were also observed in the stratified subgroup analysis, which included the analysis by ethnic subgroup (Asian and Caucasian), chemotherapy regimen (taxane-based and vinorebine-based), TUBB3 detection method (IHC and PCR), and treatment strategy (surgery plus adjuvant chemotherapy and palliative chemotherapy).

**Conclusions:**

The expression level of TUBB3 may be a useful biomarker to predict the clinical outcomes of the taxane/vinorebine-based chemotherapy in patients with NSCLC.

## Introduction

Lung cancer is the most common type of cancer and a leading cause of cancer-related death for both men and women worldwide [1]. The non-small cell lung cancer (NSCLC) accounts for approximately 85% of all lung cancer cases. Two-thirds of NSCLC cases are diagnosed at advanced stages because the patients are typically asymptomatic at early stages [2]. Due to the recent advancements in the surgical techniques and chemoradiation therapy, the one-year relative survival rate for lung cancer has increased from 35.7% to 44.5% [3]. However, the prognosis of lung cancer, especially for the advanced stage of NSCLC, is still poor. The high morbidity and mortality rates of lung cancer has continued to be a major public health concern worldwide [4].

Approximately half of NSCLC patients receive chemotherapy as part of their treatments, indicating that chemotherapy has become a common treatment method for NSCLC [5]. However, chemoresistance has emerged to be a major problem that has greatly limited the benefits of chemotherapy in patients with NSCLC [5], [6]. The clinical outcomes of chemotherapy are usually very heterogeneous and unpredictable even in NSCLC patients with similar clinical and pathologic features [7]. In addition, chemoresistance results in a waste of public health budget and makes patients suffer from unnecessary adverse effects of chemotherapy [8]. Since genetic factors may play an important role in the development of chemoresistance, it would be of great value to identify useful biomarkers that can predict the clinical outcomes of chemotherapy in NSCLC [8].

Tubulin-binding agents (TBAs), such as taxanes (paclitaxel, docetaxel) and vinca alkaloids (vinorelbine, vincristine), have been widely used in the treatment of NSCLC [9]–[11]. These agents block cell division by inhibiting the mitotic spindles. Previous *in vitro* studies have shown that high level of class III β-tubulin (TUBB3) expression was associated with chemoresistance to paclitaxel, docetaxel, and vinblastine [12]. On the basis of these preclinical results, several studies have investigated the clinical role of TUBB3 in various human cancers, mostly in NSCLC [12]–[15]. To date, a large number of clinical studies have examined the relationship between the expression of TUBB3 and the clinical outcomes of taxane/vinorebine-based chemotherapy in patients with NSCLC. However, the results were still inconclusive [16]–[43]. Some studies showed a significant association between TUBB3 and taxane/vinorebine-based chemotherapy, while the others found no correlation. Considering the relatively small sample size and the limited statistical power of an individual study, it is necessary to conduct a comprehensive up-to-date meta-analysis to evaluate the association between the expression of TUBB3 and the clinical outcomes of chemotherapy, such as the objective response rate (ORR), overall survival (OS), and event-free survival (EFS) in NSCLC patients receiving the taxane/vinorebine-based chemotherapy.

## Materials and Methods

### Literature search strategy

Relevant studies published before August 2013 were retrieved from online databases, including the PubMed, EMBASE, and China National Knowledge Infrastructure (CNKI) using the following terms and combinations: “class III β-tubulin” or “tubulin” or “TUBB3” or “lung cancer” or “neoplasm, lung”. The search was limited to full-text papers written in English or Chinese. Furthermore, the references of the retrieved studies were manually screened for additional relevant studies. If necessary, the authors of the original articles were contacted for additional data, such as the relationship between the expression level of TUBB3 and the ORR, OS, and EFS.

### Inclusion and exclusion criteria

Eligible studies were identified according to the following criteria: (1) human-based investigations; (2) pathologically confirmed non-small cell lung cancer; (3) articles published in English or Chinese; (4) taxane/vinorebine-based chemotherapy treatment; (5) investigation of the association between the expression of class III β-tubulin and the clinical outcomes of chemotherapy, including the ORR, OS, and EFS (including progression-free survival (PFS), disease-free survival (DFS), and recurrence-free survival (RFS)); (6) the full-text of the published articles were available. The exclusion criteria were as the follows: (1) patients younger than 18 years old; (2) studies in which necessary data were not provided; (3) for overlapped studies, the studies with a smaller dataset were excluded.

### Data extraction

Two investigators (YL Yang and XP Luo) independently extracted the following information from the qualified publications: surname of the first author, publication year, country, ethnicity, sample size, disease stage, ECOG performance status, chemotherapy regimen, detection method of TUBB3, and clinical outcome (ORR, OS, or EFS). All data were then examined by two investigators independently (YL Yang and XP Luo). Disagreements between the investigators were resolved by discussion. When necessary, a third investigator (L Xian) helped to reach a consensus with all investigators.

### Quality Assessment

The quality of the methodology of the included studies was assessed by the Newcastle-Ottawa scale (NOS) recommended by the Cochrane Non-Randomized Studies Methods Working Group [44]. Studies with five or more stars were defined as high quality studies. Quality assessment was performed by two investigators (YL Yang and XP Luo) independently. Disagreements were resolved by discussion.

### Statistical analysis

The ORR was assessed by the Response Evaluation Criteria In Solid Tumors (RECIST) criteria [45] or the World Health Organization (WHO) criteria (Good response  =  complete response + partial response; poor response  =  stable disease + progressive disease) [46]. The HRs and their 95% CIs for the OS and EFS were directly extracted from reports, or indirectly estimated from the Kaplan-Meier curves, or calculated by methods described by Tierney [47]. The association between the expression of TUBB3 and the clinical responses to the taxane/vinorebine-based chemotherapy was evaluated by the odds ratio (OR) with 95% CIs in the following comparison: high or positive expression of TUBB3 vs. low or negative expression of TUBB3. For the OS and EFS, the pooled HRs and 95% CIs were calculated from the HRs and 95% CIs extracted from each eligible study. Heterogeneity between studies was detected by the Q test and the I^2^ metric (no heterogeneity: I^2^ = 0%–25%; moderate heterogeneity: 25%–50%; large heterogeneity: 50%–75%; and extreme heterogeneity: 75%–100%) [48]. A fixed effect model analysis was performed when *p*≥0.10 in the Q test or when I^2^<50% [49], otherwise a random effect model analysis was conducted [50]. Subgroup analyses by ethnicity (Asian or Caucasian), chemotherapy regimen (taxane alone, vinorebine alone, or taxane and vinorebine combined), TUBB3 detection method (IHC, PCR, or Western blot), and treatment strategy (surgery plus adjuvant chemotherapy or palliative chemotherapy) were also performed. Publication bias were tested by the Begg's funnel plot [51] and the Egger's test [52]. All *p* values were two-tailed and a *p* value less than 0.05 was considered statistically significant. Most of the statistical analyses in this study were conducted by the STATA software (version 11.2; StataCorp, College Station, Texas USA).

## Results

### Eligible Studies

**Figure 1:**
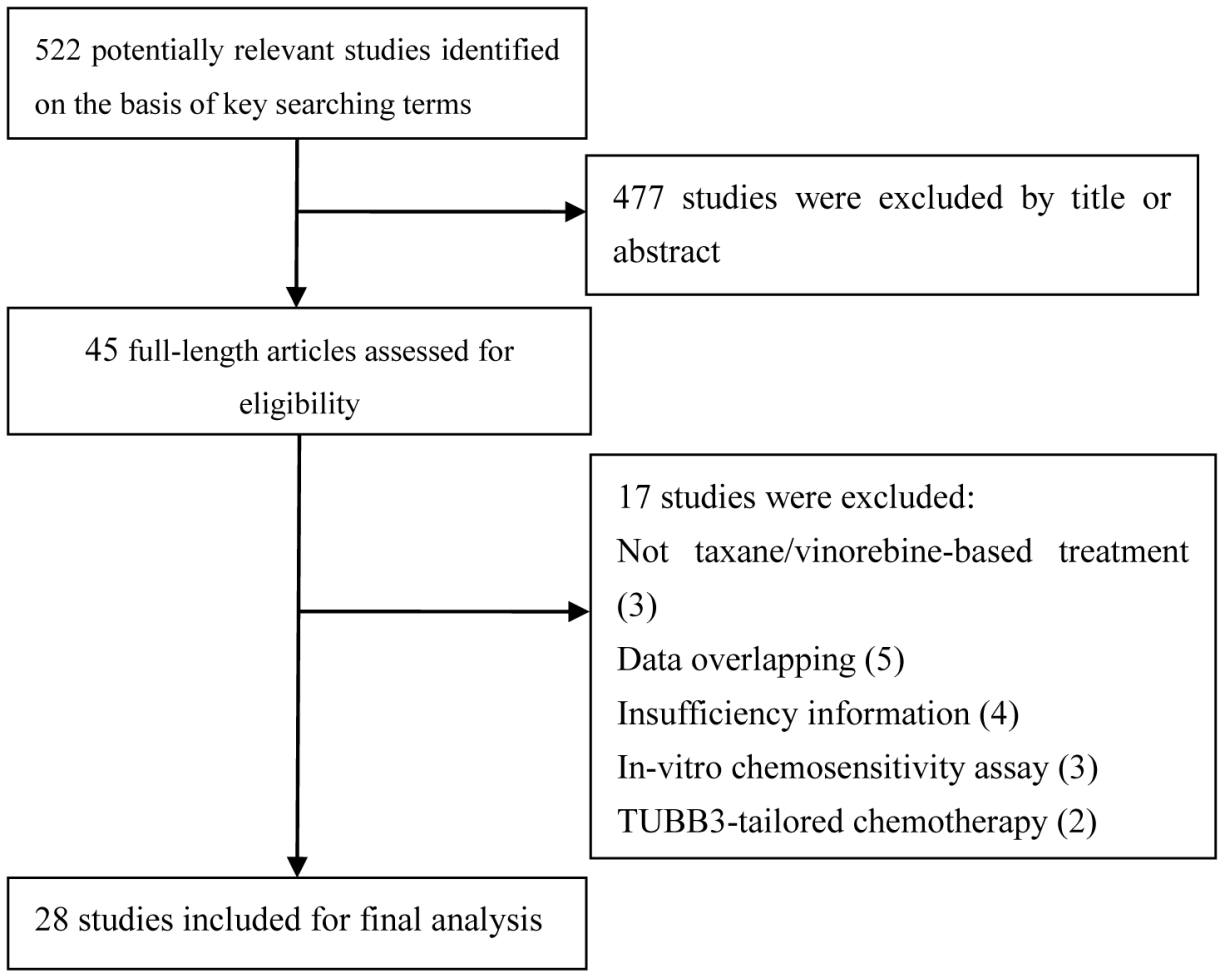
Flow diagram of the literature search in this meta-analysis.

A total of 522 studies were identified in the initial search from the three databases (PubMed, EMBASE, and CNKI). The detailed procedure of literature search was shown in Figure.1. After examining the titles and abstracts, 477 articles were excluded because they were irrelevant to this meta-analysis. The full texts of the rest 45 potentially relevant studies were carefully reviewed. As a result, 17 out of the 45 studies were excluded because of the following reasons: not taxane/vinorebine-based treatment (n = 3), data overlapping (n = 5), insufficient information (n = 4), *in vitro* chemosensitivity assay (n = 3), and TUBB3-tailored chemotherapy (n = 2). Therefore, 28 studies including 2401 patients were qualified for this meta-analysis [16]–[43]. The baseline characteristics of all eligible studies were reported in Table 1. The patient sample size of the included studies ranged from 19 to 577. There were 9 studies conducted in the Caucasian population [16]–[20], [27]–[30]; 19 studies were conducted in the Asian population [21]–[26], [31]–[43]; and two studies were conducted in multiple ethnicities. Several methods were used to assess the expression of TUBB3: immunohistochemistry (IHC) in 25 studies [16]–[30], [32]–[34], [36]–[42], polymerase chain reaction (PCR) in two studies [16], [32], and Western blot in one study [36] (Table 1). All relevant studies were assessed by the NOS quality scale and all eligible studies scored highly (with five stars or more). The quality score of the eligible studies can be found in Table S1.

**Table 1:** Baseline characteristics of the studies included in this meta-analysis.

First Author	Year	Country	Ethnicity	No. of cases	Stage, ECOG PS	Histology: No. of patients	Chemotherapy	TUBB3 detection	High expression (%)	Outcome
Rosell [16]	2003	Italy	Caucasian	53	IIIB-IV,NR	SQ21,AD22,LCUC6,Other4	vinorelbine/cisplatin,paclitaxel/carboplatin	PCR	22.6	ORR,PFS
Seve [19]	2005	France	Caucasian	47	IIIB-IV,NR	SQ17,AD23,LCC7	paclitaxel/cisplatin,carboplatin	IHC	53.2	ORR,OS,PFS
Seve [17]	2005	France	Caucasian	93	IIIB-IV,NR	SQ37,AD28,LCC28	vinorelbine/cisplatin, carboplatin	IHC	47.3	ORR,OS,PFS
Dumontet [18]	2005	France	Caucasian	19	NR	SQ8,AD9,Undiff2	paclitaxel/cisplatin; paclitaxel only	IHC	47.4	ORR,PFS
Seve [20]	2007	Canada	mixed	140	T1-2,N0-1,0-1	NR	vinorelbine/cisplatin	IHC	48.6	OS,RFS
Okuda [21]	2008	Japan	Asian	50	I-IV,NR	AD28,Other22	paclitaxel/cisplatin,carboplatin	IHC	46.0	OS
Azuma [22]	2009	Japan	Asian	45	I-III,0-2	SQ9,AD36	paclitaxel/carboplatin	IHC	35.6	ORR,OS,PFS
Azuma [23]	2009	Japan	Asian	34	II-III,0-2	SQ17,AD16,other1	docetaxel/cisplatin	IHC	35.3	ORR,OS,PFS
Ikeda [24]	2009	Japan	Asian	40	III-IV,NR	SQ23,AD13,LCC4	paclitaxel/carboplatin	IHC	55.0	OS
Huang [25]	2010	Japan	Asian	34	III,NR	SQ15,AD19	paclitaxel,docetaxel/carboplatin	IHC	32.4	OS
Kang [26]	2010	Korea	Asian	82	I-III,NR	SQ49,AD28,Other5	paclitaxel,docetaxel/cisplatin,carboplatin	IHC	NR	OS
Vilmar [27]	2011	Denmark	Caucasian	261	III-IV,0-2	SQ75,AD119,LCC9,Other58	paclitaxel,vinorelbine/cisplatin	IHC	42.9	ORR,OS,PFS
Reiman [28]	2012	Canada	mixed	577	I-III,0-2	NR	paclitaxel,vinorelbine/cisplatin	IHC	47.8	OS,DFS
Christoph [29]	2012	Germany	Caucasian	65	I-IV,NR	SQ16,AD29,LCC6,Other14	paclitaxel,vinorelbine/platinum	IHC	49.2	OS,PFS
Krawczyk [30]	2012	Poland	Caucasian	102	III-IV,0-2	SQ68, Other36	docetaxel	IHC	9.8	OS,PFS
Kaira [31]	2013	Japan	Asian	24	II-III,0-2	AD16,Other8	docetaxel	IHC	44.0	ORR,PFS
Jiang [32]	2013	China	Asian	73	II,NR	SQ36,AD33,Other4	vinorelbine/cisplatin	PCR	NR	OS,DFS
Zhang YZ [33]	2013	China	Asian	85	I-IIIA,NR	SQ29,AD48,Other8	vinorelbine/cisplatin,carboplatin	IHC	57.6	OS,DFS
Xiao [34]	2009	China	Asian	53	IIIB-IV,0-2	SQ25,AD27,LCC1	vinorelbine/platinum, taxane/platinum	IHC	58.5	ORR,OS
Yang [35]	2009	China	Asian	84	IV,NR	SQ43,AD41	paclitaxel/cisplatin	IHC	48.8	ORR,OS,PFS
Pu [36]	2009	China	Asian	90	IIIB-IV,0-2	SQ44,AD42,LCC2	vinorelbine/cisplatin, carboplatin	Western blot	50.0	ORR
Gong [37]	2009	China	Asian	30	II-III,NR	SQ27,AD34	Taxane based	IHC	70.0	ORR
Wan [38]	2011	China	Asian	87	IIIB-IV,0-2	SQ56,AD23,LCC8	docetaxel/cisplatin, docetaxel	IHC	48.3	ORR
Guo [39]	2011	China	Asian	33	III-IV	SQ16,AD17	paclitaxel/cisplatin, carboplatin	IHC	8/33	ORR
Zhou [40]	2012	China	Asian	64	III-IV	SQ31,AD33	paclitaxel-based	IHC	24.2	ORR
Zhang JP [41]	2012	China	Asian	63	III-IV	SQ29,AD34	paclitaxel/cisplatin	IHC	69.8	ORR
Gao [42]	2012	China	Asian	55	IIIB-IV,0-2	SQ27,AD28	paclitaxel/cisplatin	IHC	50.9	ORR
Liu [43]	2013	China	Asian	120	IIIB-IV,0-3	SQ68,AD52	vinorelbine/platinum, taxane/platinum	IHC	63.3	ORR

**Abbreviations:** **NR**, not reported; **SQ**, squamous cell cancer; **AD**, adenocarcinoma; **LCC**, large cell carcinoma; **Undiff**, undifferetiation;

**IHC**, immunohistochemistry; **PCR**, polymerase chain reaction; **PFS**, progression-free survival; **DFS**, disease-free survival; **ORR**, objective response rate; **OS**, overall survival.

### Objective response rate

**Figure 2:**
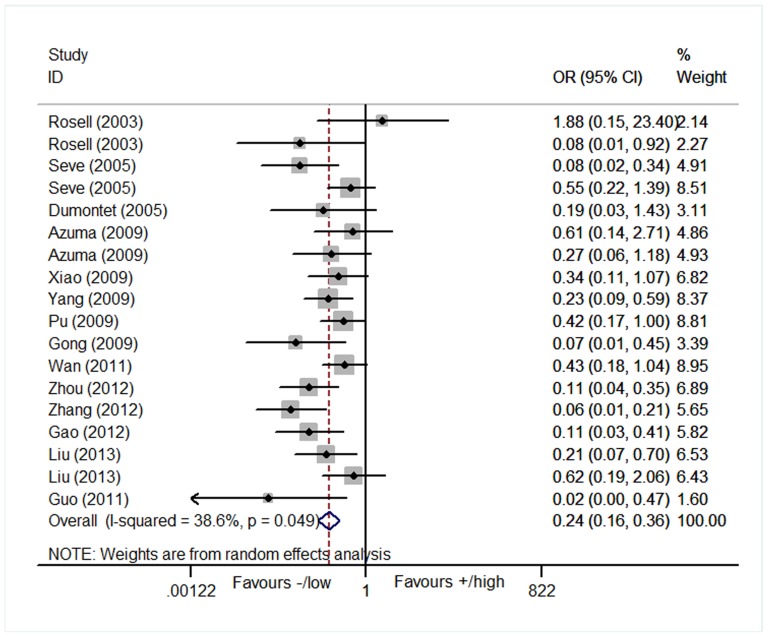
Forest plot for the association between the expression level of TUBB3 and the objective response rate (ORR) in patients receiving the taxane/vinorebine-based chemotherapy.

The association between the expression of TUBB3 and the treatment response to the taxane/vinorebine-based chemotherapy was investigated in 18 studies which consisted of 954 patients [16]–[19], [22], [23], [27], [31], [34]–[43]. We found that the positive or high level of TUBB3 expression was significantly associated with a worse response to chemotherapy when compared to the negative or low level of TUBB3 in the random model analysis (positive/high vs. negative/low: OR = 0.24, 95% CI = 0.16–0.36, *p*<0.001; I^2^ = 38.6%, P = 0.05 for heterogeneity, Table 2, Figure. 2).

**Table 2:** Meta-analysis results of TUBB3 expression and the ORR, OS, and EFS in NSCLC patients receiving the taxane/vinorelbine-based chemotherapy.

	ORR					OS					EFS			
	Study	Model	OR(95%CI),*p*	I^2^,P		Study	Model	HR(95%CI), *p*	I^2^,P		Study	Model	HR(95%CI), *p*	I^2^,P
All	18(954)	R	0.24(0.16,0.36),<0.001	38.6%,0.05		18(1879)	R	1.52(1.27,1.82),<0.001	38%,0.05		14(1640)	R	1.47(1.24,1.74),<0.001	37.0%,0.08
Ethnicity														
Asian	13(758)	R	0.23(0.15,0.36),<0.001	37.2%,0.09		11(594)	F	1.58(1.30,1.92),<0.001	14.2%,0.31		6(336)	F	1.65(1.35,2.02),<0.001	3.3%,0.40
Caucasian	5(196)	R	0.26(0.09,0.79),0.02	51.3%,0.08		7(1285)	R	1.48(1.14,1.94),0.004	55.6%,0.04		8(1304)	F	1.27(1.10,1.46),0.001	36.3%,0.14
Chemotherapy regimen														
Taxane-based	13(641)	F	0.18(0.12,0.25),<0.001	23.4%,0.21		10(533)	F	2.06(1.57,2.72),<0.001	0.0%,0.71		7(346)	F	2.05(1.55,2.71),<0.001	0.0%,0.50
Vinorebine-based	4(260)	F	0.54(0.31,0.93),0.03	0.0%,0.72		4(390)	F	1.45(1,17,1.79),0.001	37.6%,0.19		4(391)	F	1.50(1.22,1.86),<0.001	0.0%,0.67
Both	1(53)	-	0.34(0.11,1.07),0.065	-		4(956)	F	1.19(1.01,1.41),0.038	39.4%,0.18		3(903)	F	1.16(0.99,1.36),0.075	0.0%,0.67
Detection method														
IHC	15(827)	R	0.22(0.14,0.34),<0.001	39.4%,0.06		16(1704)	R	1.55(1.25,1.93),<0.001	44%,0.03		11(1418)	F	1.30(1.14,1.39),<0.001	34.1%,013
PCR	2(37)	R	0.38(0.02,8.38),0.54	67.8%,0.08		2(175)	F	1.54(1.18,1.99),0.001	0.0%,0.85		3(222)	F	1.68(1.32,2.14),0.001	11.9%,0.32
WB	1(90)	-	0.42(0.17,1.00),0.05	-										
Treatment strategy														
S+chem	1(45)	-	0.61(0.14,2.71),0.51	-		7(1050)	F	1.32(1.13,1.55),0.001	43.2%,0.103		4(875)	F	1.32(1.12,1.54),0.001	0.0%,0.42
Palliative chem	17(909)	R	0.23(0.16,0.36),<0.001	39.3%,0.05		11(829)	F	1.51(1.26,1.80),<0.001	36.2%,0.11		10(765)	R	1.63(1.24,2.14),<0.001	47.1%,0.05

**Abbreviations**: **TUBB3**, β-III tubulin; **R**, random model; **F**, fixed model; **WB**, western blot; **IHC**, immunohistochemistry; **PCR**, polymerase chain reaction; **ORR**, objective response rate; **OS**, overall survival; **EFS**, event free survival; **S+chem**, surgery plus adjuvant chemotherapy; **Palliative chem**, palliative chemotherapy.

Stratified subgroup analysis by ethnicity, chemotherapy regimen, and TUBB3 detection method were also performed in this meta-analysis. There were 13 investigations for the Asian population that were comprised of 758 cases [22], [23], [31], [34]–[43]. In addition,five studies composed of 196 cases were conducted in the Caucasian population [16]–[19], [27]. Ethnic subgroup analysis indicated that the negative or low level of TUBB3 expression was correlated with a better chemotherapy response in both Asian (OR = 0.23, 95% CI = 0.15–0.36, *p*<0.001; I^2^ = 37.2%, P = 0.09 for heterogeneity) and Caucasian patients (OR = 0.26, 95% CI = 0.09–0.79, *p* = 0.02; I^2^ = 51.3%, P = 0.08 for heterogeneity, Table 2).

In the subgroup analysis by the chemotherapy regimens, 13 studies used the taxane-based regimens [16], [18], [19], [22], [23], [35], [37]–[43], four studies investigated the vinorebine-based regimens [16], [17], [36], [43], and one study examined both taxane and vinorebine-based regimens [34]. The studies by Rosell [16] and Liu [43] reported both the patients receiving the taxane-based regimen and the vinorebine-based regimen. The study by Xiao [34], however, did not provide sufficient information to distinguish the patients receiving the taxane-based chemotherapy from the patients receiving the vinorebine-based chemotherapy. Our analysis suggested that the negative or low level of TUBB3 expression showed a better response than the positive or high level of TUBB3 expression in the taxane-based regimen (positive/high vs. negative/low: OR = 0.18, 95% CI = 0.12–0.25, *p*<0.001; I^2^ = 23,4%, P = 0.21 for heterogeneity) and the vinorebine-based regimen (positive/high vs. negative/low: OR = 0.54, 95% CI = 0.31–0.93, *p* = 0.03; I^2^ = 0.0%, P = 0.72 for heterogeneity). However, these associations were not found in Xiao's study, in which the analysis results were based on a mixed patient population receiving either the taxane- or vinorebine-based regimens (positive/high vs. negative/low: OR = 0.34, 95% CI = 0.11–1.07, p = 0.065, Table 2) [34].

In this study, 14 studies used IHC [17]-[19], [22], [23], [34], [35], [37]-[41], [43], one study used PCR [16], and one study used Western Blot [36] to detect the expression of TUBB3. Only the studies using IHC showed a significant association between the expression of TUBB3 and the ORR in patients with NSCLC (OR = 0.22, 95% CI = 0.14–0.34, *p*<0.001; I^2^ = 39.4%, P = 0.06 for heterogeneity, Table 2).

Except for the study by Azuma K et al. that reported patients receiving surgery plus adjuvant chemotherapy [22], the rest of the studies reported patients receiving palliative chemotherapy [16]–[19], [23], [27], [31], [34]–[43]. Our analysis suggested that the patients with negative or low level of TUBB3 expression had a better treatment response than those with positive or high level of TUBB3 expression in the palliative taxane/vinorebine-based chemotherapy (OR = 0.23, 95% CI = 0.14–0.37, p<0.001; I^2^ = 46.7%, P = 0.04 for heterogeneity, Table 2).

### Overall survival

**Figure 3:**
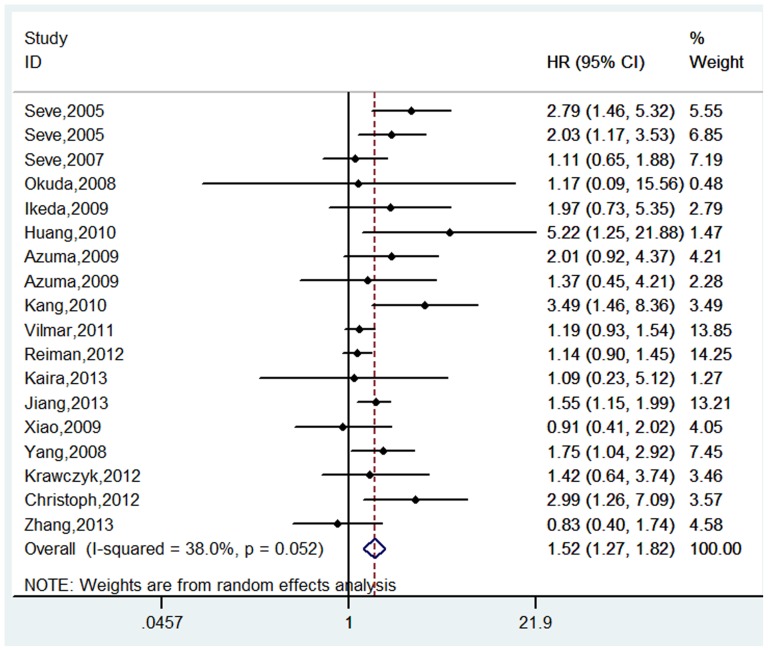
Forest plot for the association between the expression level of TUBB3 and the overall survival (OS) in patients receiving the taxane/vinorebine-based chemotherapy.

18 studies including 1879 patients evaluated the association between the expression of TUBB3 and the OS in NSCLC patients receiving the taxane/vinorebine-based chemotherapy [17], [19]–[35]. The pooled analysis indicated that the patients with positive or high level of TUBB3 had a shorter OS than the patients with negative or low level of TUBB3 (positive/high vs. negative/low: HR = 1.52, 95% CI = 1.27–1.82, *p*<0.001; I^2^ = 38.0%, P = 0.05 for heterogeneity, Table 2, Figure 3).

In the stratified analysis by ethnicity, we found that the expression of TUBB3 was correlated with the OS in both Asian [21]–[26], [31]–[35] (HR = 1.48, 95% CI = 1.21–1.82, *p*<0.001; I^2^ = 13.2%, P = 0.31 for heterogeneity) and Caucasian patients [17], [19], [20], [27]–[30] (HR = 1.48, 95% CI = 1.14–1.92, *p* = 0.004; I^2^ = 55.6%, P = 0.04 for heterogeneity, Table 2). In the subgroup analysis by chemotherapy regimen, ten studies investigated the taxane-based regimens [19], [21]–[23], [25], [26], [30], [31], [35], [53]; four reported the vinorebine-based regimens [17], [20], [32], [33]; and four studies examined both regimens [27]–[29], [34]. The results of our analysis showed that the negative or low TUBB3 expression was associated with a longer OS than the positive or high TUBB3 expression in NSCLC patients receiving the taxane-based, or vinorebine-based, or both taxane- and vinorebine-based regimens (taxane regimen: HR = 2.06, 95% CI = 1.57–2.72, *p*<0.001; I^2^ = 0.0%, P = 0.71 for heterogeneity; vinorebine regimen: HR = 1.45, 95% CI = 1.17–1.79, *p* = 0.001; I^2^ = 37.6%, P = 0.19 for heterogeneity; taxane and vinorebine combined regimen: HR = 1.19, 95% CI = 1.01–1.41, *p* = 0.038; I^2^ = 39.4%, P = 0.18 for heterogeneity, Table 2). The subgroup analysis of the TUBB3 detection methods indicated that the expression of TUBB3 was significantly associated with the OS in the 16 studies using IHC as the detection method for TUBB3 [17], [19]–[23], [25]–[30], [33], [35], [53] (HR = 1.55, 95% CI = 1.25–1.93, *p*<0.001; I^2^ = 44.0%, P = 0.03 for heterogeneity), as well as in the two studies using PCR [30], [32] (HR = 1.54, 95% CI = 1.18–1.99, *p* = 0.001; I^2^ = 0.0%, P = 0.85 for heterogeneity, Table 2). Additional stratified analysis based on the treatment strategy showed that the expression of TUBB3 could be used as a prognostic biomarker to predict the clinical outcomes in patients treated by surgery plus adjuvant chemotherapy [20]–[22], [26], [28], [32], [33] (HR = 1.32, 95% CI = 1.13–1.55, *p* = 0.001; I^2^ = 0.0%, P  =  0.71 for heterogeneity) and patients treated by palliative chemotherapy [17], [19], [23]–[25], [27], [29]–[31], [34], [35] (HR = 1.51, 95% CI = 1.26–1.80, *p*<0.001; I^2^ = 36.2%, P = 0.11 for heterogeneity, Table 2)

### Event free survival

**Figure 4:**
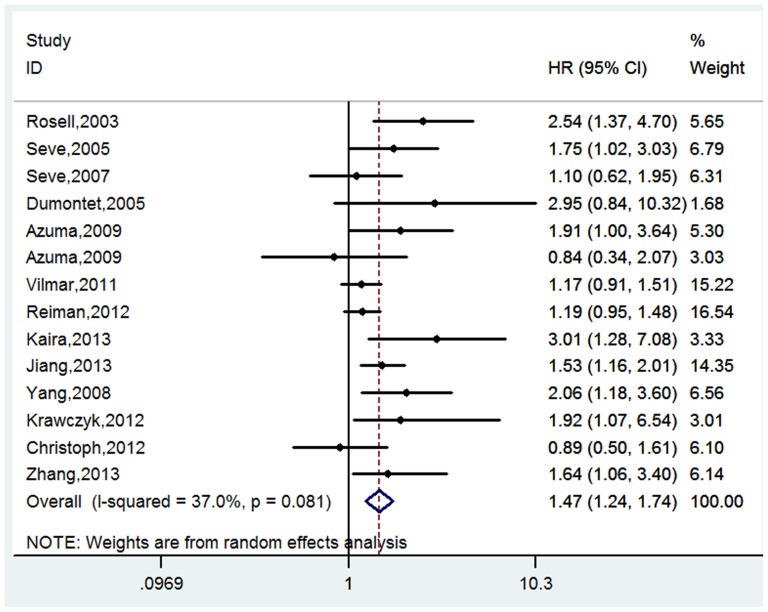
Forest plot for the association between the expression level of TUBB3 and the event-free survival (EFS) in patients receiving the taxane/vinorebine-based chemotherapy.

A total of 14 studies composed of 1640 patients reported the event free survival (EFS) in this meta-analysis [16]–[20], [22], [23], [27]–[33], [35]. Among them, one study used the RFS as its endpoint [20], three studies examined the DFS [28], [32], [33], and the rest ten studies evaluated the PFS [16]–[19], [22], [23], [27], [29]–[31], [35]. There was strong evidence to support that the expression of TUBB3 was correlated with the EFS (positive/high vs. negative/low: HR = 1.47, 95% CI = 1.24–1.74, *p*<0.001; I^2^ = 37.0%, P = 0.08 for heterogeneity, Table 2, Figure 4).

The stratified analysis by ethnicity showed that the association between TUBB3 and EFS was significant in both Asian [22], [23], [31]–[33], [35] and Caucasian populations [16], [17], [20], [27]–[30] (Asian: HR = 1.65, 95% CI = 1.35–2.02, *p*<0.001; I^2^ = 3.3%, P = 0.40 for heterogeneity; Caucasian: HR = 1.27, 95% CI = 1.10–1.46, *p* = 0.001; I^2^ = 36.3%, P = 0.14 for heterogeneity, Table 2). Among the 14 studies that reported data on EFS, eight studies used the taxane-based regimen [16], [18], [19], [22], [23], [30], [31], [35], four studies examined the vinorebine-based regimen [17], [20], [32], [33], and three studies investigated both regimens [27]–[29]. The stratified analysis by chemotherapy regimen suggested that the positive or high expression of TUBB3 was correlated with a worse EFS in patients receiving the taxane-based or vinorebine-based regimens, but not in patients treated by the taxane and vinorebine combined regimen (taxane regimen: HR = 2.05, 95% CI = 1.55–2.71, *p*<0.001; I^2^ = 0.0%, P = 0.50 for heterogeneity; vinorebine regimen: HR = 1.50, 95% CI = 1.22–1.86, *p*<0.001; I^2^ = 0.0%, P = 0.67 for heterogeneity; taxane and vinorebine combined regimen: HR = 1.16, 95% CI = 0.99–1.36, *p* = 0.075; I^2^ = 0.0%, P = 0.67 for heterogeneity, Table 2). In the stratified analysis by TUBB3 detection method, three studies used PCR to detect TUBB3 expression [16], [30], [32] and the remaining 11 studies chose IHC to assess TUBB3 expression [17]–[20], [22], [23], [27]–[29], [31], [33], [35]. Our analysis showed that the expression of TUBB3 was significantly associated with the EFS in both groups (PCR: HR = 1.30, 95% CI = 1.14–1.39, *p*<0.001; I^2^ = 34.1%, P = 0.13 for heterogeneity; IHC: HR = 1.68, 95% CI = 1.32–2.14, *p*<0.001; I^2^ = 11.9%, P = 0.32 for heterogeneity, Table 2). In the subgroup analysis by treatment strategy, four studies evaluated the surgery plus adjuvant chemotherapy [20], [28], [32], [33] and ten studies reported results from the palliative chemotherapy [16]–[18], [22], [23], [27], [29]–[31], [35]. Our analysis showed that the patients with negative or low level of TUBB3 had a longer EFS than the patients with positive or high level of TUBB3 (surgery plus adjuvant chemotherapy: HR = 1.32, 95% CI = 1.12–1.54, *p* = 0.001; I^2^ = 0.0%, P = 0.42 for heterogeneity; palliative chemotherapy: HR = 1.63, 95% CI = 1.24–2.14, *p*<0.001; I^2^ = 47.1%, P = 0.05 for heterogeneity, Table 2).

### Publication bias

Publication bias was evaluated by the funnel plots qualitatively and tested by the Begg's and the Egger's tests quantitatively. Visual inspection of the Begg's funnel plots did not show significant evidences of asymmetry in the ORR, OS, and EFS (figure not shown), suggesting no publication bias in this meta-analysis. In addition, no significant bias was found in the Begg's test and the Egger's test. For the Begg's test, P was 0.17, 0.48, and 0.16 for the ORR, OS, and EFS respectively; for the Egger's test, P was 0.20, 0.07, and 0.06 for the ORR, OS, and EFS respectively (figure not shown).

## Discussion

In this meta-analysis, we explored the predictive role of TUBB3 in patients receiving the taxane/vinorebine-based chemotherapy. We found that the high level of TUBB3 expression was associated with a lower objective response rate (ORR), a shorter overall survival (OS), and a worse event-free survival (EFS) compared to the low level of TUBB3 expression. Consistently, a previous meta-analysis by Zhang et al. [54] reported that TUBB3 was a biomarker for the sensitivity of paclitaxel/vinorebine-based chemotherapy in patients with NSCLC.

We think it is necessary to conduct an updated meta-analysis to re-evaluate the association between the expression of TUBB3 and the efficacy of taxane/vinorebine-based chemotherapy for the following reasons. First of all, the latest studies included in the previous meta-analysis by Zhang et al. [54] were published before 2009. There have been a number of studies with large patient sample size being published since 2009. In fact, our study has a much larger sample size (2401 patients from 28 studies) compared to the study by Zhang et al. (552 patients from 10 studies), which gives more reliable results in our analysis. In addition, Zhang and colleagues only examined the objective response rate and the median survival time in their analysis. In our study, we used the objective response rate, overall survival, and event free survival as the primary parameters to assess the association between the expression of TUBB3 and the clinical outcomes of taxane/vinorebine-based chemotherapy. Because a low ORR suggests tumor resistance to the chemotherapy regimen and a short OS/EFS indicates a poor prognosis, it is necessary to include all three parameters in order to make a comprehensive assessment about the treatment response to a chemotherapy regimen [55]. In our analysis, we demonstrated that the high level of TUBB3 expression was associated with a lower ORR, a shorter OS, and a worse EFS, which strongly supported that TUBB3 had a prognostic value in predicting the treatment response to the taxane/vinorebine-based chemotherapy. Furthermore, we performed a subgroup analysis by the TUBB3 detection method and treatment strategy, which were not done in the study by Zhang and colleagues.

Microtubule is composed of polymers of tubulin dimers and plays an important role in the development and maintenance of cell polarity, vesicle and organelle transportation, cellular signaling, and cell division. Tubulin and microtubules are the main targets of the vinca alkaloids. Vinorelbine binds to the β-subunit of tubulin dimers at a distinct region called the vinca-binding domain. In contrast, paclitaxel binds to β-tubulin within the lumen of microtubule. This binding event affects a protein loop, called the M-loop, which is thought to stabilize the lateral interactions between the adjacent protofilaments of microtubule [12], [57]. Binding of taxane/vinorebine inhibits the association/dissociation of tubulin dimers in microtubule and thus disrupts the spindle dynamics, resulting in cell cycle arrest in the transition from metaphase to anaphase and eventually causes apoptotic cell death [56]. However, the molecular mechanism underlying the association between the expression level of TUBB3 and the sensitivity of taxane/vinorebine chemotherapy remains an unanswered question. Further studies are needed to address this question.

Our meta-analysis showed that the overexpression of TUBB3 may be an important factor for the development of chemoresistance to taxane/vinorebine. Hence, the expression level of TUBB3 may serve as a useful biomarker in the individually-tailored personalized chemotherapy in the future. The patient with low level of TUBB3 may benefit from the taxane/vinorebine-based regimens, whereas those with high level of TUBB3 should avoid the taxane or vinorebine-based chemotherapy and replace them with chemotherapy drugs that can overcome chemoresistance, such as ixabepilone [57].

There are some limitations in our meta-analysis. Firstly, the studies included in this meta-analysis had different study designs, such as the patient selection criteria and chemotherapy protocols. Some studies included radiotherapy in addition to the chemotherapy while the others did not. These differences could contribute to the heterogeneity among those studies.

Secondly, some factors such as age, sex, smoking history, histology type, tumor stage, and treatment method may affect the prognosis in patients receiving chemotherapy treatment. However, we could not conduct a stratified analysis to assess the effects of confounding factors on the predictive role of TUBB3 in NSCLC patients because of the limited information provided in the original publications.

Moreover, the HRs and their 95% CI we extracted from the time-to-event data were not consistent. We have to estimate the HRs by reading the Kaplan-Meier curves because some studies did not report the HRs. Some studies reported the unadjusted HRs while the others provided the adjusted HRs. Moreover, the cofounders they adjusted were not the same for the adjusted HRs. All of these factors more or less contributed to the heterogeneity in this study.

At last, potential publication biases may exist. Articles were not written in English or Chinese and studies failed to get published because of negative or null results cannot be identified in our literature search and thus were not included in this analysis. In addition, some reports did not provide sufficient data were also excluded from our analysis.

In conclusion, our meta-analysis indicated that the expression of TUBB3 may be a useful biomarker to predict the clinical outcomes of taxane/vinorebine-based chemotherapy in patients with NSCLC. With the limitations, heterogeneities, and bias of meta-analysis, our conclusions in this study need to be interpreted with caution. Future large prospective studies with rigorously designed methodology are warranted to confirm our results.

## Supporting Information

Checklist S1 PRISMA checklist.
(DOC)

Table S1 Quality assessment of eligible studies by the Newcastle-Ottawa Scale.
(DOCX)
