# Supplementary material for: The Prognostic Role of the Class III β-Tubulin in Non-Small Cell Lung Cancer (NSCLC) Patients Receiving the Taxane/Vinorebine-Based Chemotherapy: A Meta-Analysis
Source: PLoS One. 2014 Apr 4;9(4):e93997. doi: 10.1371/journal.pone.0093997 (PMC3976369; doi:10.1371/journal.pone.0093997)
Supplement: Table S1 — Quality assessment of eligible studies by the Newcastle-Ottawa Scale. (DOCX) [file pone.0093997.s002.docx]

Table S2.Quality assessment of eligible studies with Newcastle-Ottawa Scale

| Author | Year | Selection | Comparability | Outcome |
| --- | --- | --- | --- | --- |
| Rosell | 2003 | ★★★ | ★★ | ★★★ |
| Seve | 2005 | ★★★★ | ★★ | ★★★ |
| Seve | 2005 | ★★★★ | ★★ | ★★★ |
| Dumontet | 2005 | ★★★ | ★★ | ★★★ |
| Seve | 2007 | ★★★★ | ★★ | ★★★ |
| Okuda | 2008 | ★★★ | ★★ | ★★★ |
| Azuma | 2009 | ★★★★ | ★★ | ★★★ |
| Azuma | 2009 | ★★★★ | ★★ | ★★★ |
| Ikeda | 2009 | ★★★ | ★★ | ★★★ |
| Huang | 2010 | ★★★★ | ★★ | ★★★ |
| Kang | 2010 | ★★★★ | ★★ | ★★★ |
| Vilmar | 2011 | ★★★★ | ★★ | ★★★ |
| Reiman | 2012 | ★★★★ | ★★ | ★★★ |
| Christoph | 2012 | ★★★★ | ★★ | ★★ |
| Krawczyk | 2012 | ★★★ | ★★ | ★★★ |
| Kaira | 2013 | ★★★ | ★★ | ★★ |
| Jiang | 2013 | ★★★★ | ★★ | ★★★ |
| Zhang YZ | 2013 | ★★★ | ★★ | ★★★ |
| Xiao | 2009 | ★★★★ | ★ | ★★★ |
| Yang | 2009 | ★★★ | ★★ | ★★★ |
| Pu | 2009 | ★★★ | ★★ | ★★★ |
| Gong | 2009 | ★★★ | ★ | ★★ |
| Wan | 2011 | ★★★★ | ★ | ★★ |
| Guo | 2011 | ★★★ | ★ | ★★ |
| Zhou | 2012 | ★★★★ | ★★ | ★★ |
| Zhang JP | 2012 | ★★★ | ★ | ★★ |
| Gao | 2012 | ★★★ | ★ | ★★ |
| Liu | 2013 | ★★★ | ★★ | ★★ |
